# Nucleolar damage correlates with neurotoxicity induced by different platinum drugs

**DOI:** 10.1054/bjoc.2001.2024

**Published:** 2001-10

**Authors:** M J McKeage, T Hsu, D Screnci, G Haddad, B C Baguley

**Affiliations:** Division of Pharmacology and Clinical Pharmacology, Faculty of Medicine and Health Sciences, The University of Auckland, Auckland, New Zealand; Auckland Cancer Society Research Centre, Faculty of Medicine and Health Sciences, The University of Auckland, Auckland, New Zealand

**Keywords:** platinum drugs, cancer chemotherapy, neurotoxicity, dorsal root ganglia, nucleolus

## Abstract

Platinum-based drugs are very useful in cancer therapy but are associated with neurotoxicity in the clinic. To investigate the mechanism of neurotoxicity, dorsal root ganglia of rats treated with various platinum drugs were studied. Cell body, nuclear and nucleolar dimensions of dorsal root ganglia sensory nerve cells were measured to determine morphological toxicity. Sensory nerve conduction velocity was measured to determine functional toxicity. After a single dose of oxaliplatin (10 mg kg^−1^), no significant change in nuclear and cell body diameter was seen but decreased nucleolar size was apparent within a few hours of treatment. Changes in nucleolar size were maximal at 24 hours, recovered very slowly and showed a non-linear dependence on oxaliplatin dose (r^2^= 0.99). Functional toxicity was delayed in onset until 14 days after a single dose of oxaliplatin but eventually recovered 3 months after treatment. Multiple doses of cisplatin, carboplatin, oxaliplatin, *R*, *R* -ormaplatin and *S*, *S* -ormaplatin were also associated with time-dependent reduction in nucleolar size. A linear correlation was obtained between the rate of change in nucleolar size during multiple dose treatment with the series of platinum drugs and the time taken for the development of altered sensory nerve conduction velocity (r^2^= 0.86;*P*< 0.024). Damage to the nucleolus of ganglionic sensory neurons is therefore linked to the neurotoxicity of platinum-based drugs, possibly through mechanisms resulting in the inhibition of rRNA synthesis. © 2001 Cancer Research Campaign  http://www.bjcancer.com


*British Journal of Cancer* (2001) **85**(8), 1219–1225
© 2001 Cancer Research Campaign
doi: 10.1054/ bjoc.2001.2024, available online at http://www.idealibrary.com on

### Nucleolar damage correlates with neurotoxicity induced by different platinum drugs

**MJ McKeage[1], T Hsu[1], D Screnci[1], G Haddad[1] and BC Baguley[2]**

[1]Division of Pharmacology and Clinical Pharmacology, Faculty of Medicine and Health Sciences, The University of Auckland, Auckland, New Zealand;
[2]Auckland Cancer Society Research Centre, Faculty of Medicine and Health Sciences, The University of Auckland, Auckland, New Zealand

**Summary** Platinum-based drugs are very useful in cancer therapy but are associated with neurotoxicity in the clinic. To investigate the mechanism of neurotoxicity, dorsal root ganglia of rats treated with various platinum drugs were studied. Cell body, nuclear and nucleolar dimensions of dorsal root ganglia sensory nerve cells were measured to determine morphological toxicity. Sensory nerve conduction velocity was measured to determine functional toxicity. After a single dose of oxaliplatin (10 mg $kg^{-1}$), no significant change in nuclear and cell body diameter was seen but decreased nucleolar size was apparent within a few hours of treatment. Changes in nucleolar size were maximal at 24 hours, recovered very slowly and showed a non-linear dependence on oxaliplatin dose ($r^2 = 0.99$). Functional toxicity was delayed in onset until 14 days after a single dose of oxaliplatin but eventually recovered 3 months after treatment. Multiple doses of cisplatin, carboplatin, oxaliplatin, *R*, *R*-ormaplatin and *S*, *S*-ormaplatin were also associated with time-dependent reduction in nucleolar size. A linear correlation was obtained between the rate of change in nucleolar size during multiple dose treatment with the series of platinum drugs and the time taken for the development of altered sensory nerve conduction velocity ($r^2 = 0.86$; $P < 0.024$). Damage to the nucleolus of ganglionic sensory neurons is therefore linked to the neurotoxicity of platinum-based drugs, possibly through mechanisms resulting in the inhibition of rRNA synthesis. © 2001 Cancer Research Campaign http://www.bjcancer.com

**Keywords**: platinum drugs; cancer chemotherapy; neurotoxicity; dorsal root ganglia; nucleolus

Platinum-based drugs are widely used in cancer chemotherapy and have been associated with improved cure rates and survival-time of patients with testicular (Einhorn, 1998), cervical (Thomas, 1999) and ovarian cancer (Ozols, 2000). Platinum-based drugs are also associated with a number of side effects in the clinic that impede their application in cancer therapy. Considerable progress has been made in the clinical management of acute emesis, thrombocytopenia and nephrotoxicity (reviewed in McKeage, 2000). Peripheral neurotoxicity remains a significant problem and the dose-limiting toxicity for cisplatin (Ozols et al, 1988), oxaliplatin (Extra et al, 1998; Misset, 1998) and ormaplatin (Schilder et al, 1994; O'Rourke et al, 1998). In the form of sensory neuropathy and ototoxicity, this toxicity remains a major obstacle to the clinical application of platinum drugs.

Clinical features of the neurotoxicity suggest that dorsal root ganglia are a site of toxicity (Thompson et al, 1984). Dorsal root ganglia are discrete collections of nervous tissue located near the spine and contain the cell bodies of the peripheral sensory nerve cells. Dorsal root ganglia have no blood–brain barrier and accumulate high levels of platinum drugs compared to tissues such as brain and spinal cord that are protected by the barrier (Thompson et al, 1984; Gregg et al, 1992; Screnci et al, 1997, 2000). Morphological changes have been documented in dorsal root ganglia after platinum drug treatment involving these sensory nerve cells (Tomiwa et al, 1986; Muller et al, 1990; Cavaletti et al, 1992, 1998; Cece et al, 1995a; Stacchiotti et al, 1995; Holmes et al, 1998). Changes in ganglion-supporting cells appear simply reactive to the damaged neurons (Cece et al, 1995b). The mechanism of neurotoxicity therefore appears to involve damage to sensory nerve cells within dorsal root ganglia, but exactly how the damage comes about is unclear.

The biological target of platinum drugs involved in their cancer chemotherapeutic activity is widely regarded to be DNA. Platinum drugs react with DNA forming intra-strand and inter-strand cross-links, and distorting the DNA template (Jamieson and Lippard, 1999). The DNA damage caused by platinum drugs inhibits the replication of DNA, an effect considered to play a possible role in their action against replicating tumour cells (Harder and Rosenberg, 1970). Their mode of action has also been proposed to involve the generation of cellular signals in response to the detection of DNA damage, leading to cell cycle arrest and the induction of programmed cell death (Sorenson et al, 1992; Fink and Howell, 2000). Neither mechanism readily explains the neurotoxicity of platinum drugs since the sensory neuron cells contained within dorsal root ganglia are post-mitotic cells and therefore are not carrying out DNA replication or moving through the cell cycle.

To investigate the mechanism of neurotoxicity associated with platinum drugs, we studied the dorsal root ganglia of rats after treatment with platinum drugs to determine the time course of histological toxicity and the relationship to functional neurotoxicity. Changes to the nucleoli of dorsal root ganglia sensory nerve cells occurred very early after platinum drug treatment and correlated with the subsequent development of functional neurotoxicity as determined by changes in nerve conduction.

*Received 28 March 2001*
*Revised 19 June 2001*
*Accepted 21 June 2001*

*Correspondence to:* MJ McKeage

### METHODS

### Drugs

Cisplatin and carboplatin were purchased from Sigma (St Louis, MO, USA). Oxaliplatin and the *R*, *R*-and *S*, *S*-enantiomers of ormaplatin were synthesised as outlined previously (Screnci et al, 1997).

### Animals and treatment

Age-matched outbred female Wistar rats were used. Animals were acclimatised to handling for 2 weeks prior to the start of the experiment. The animals were 10 weeks old and weighed between 200 and 250 grams at the start of the experiment. Solutions of platinum drugs were prepared in 0.9% (w/v) NaCl (Baxter Healthcare, New Zealand) by vortex mixing and sonication at an injection volume of 10 ml $kg^{-1}$ body weight. The platinum drugs were administered by intraperitoneal injection. Control animals received the drug vehicle alone. The University of Auckland Animal Ethics Committee approved the project and the animal ethics meet the requirements of the UKCCCR guidelines. Animals had continuous access to food and water. Animals were weighed and checked for signs of drug toxicity at least twice per week. Any animals showing signs of distress were immediately and painlessly killed.

### Dorsal root ganglia morphology

Animals were first deeply anaesthetised with sodium pentobarbitone (Chemostock Animal Health Ltd, Christchurch, New Zealand) at a dose of 90 mg $kg^{-1}$. Intra-cardiac paraformaldehyde perfusion was carried out by giving 60 ml of 0.9% sodium chloride followed by 60 ml of 4% paraformaldehyde. L5 dorsal root ganglia were dissected out and placed in 4% paraformaldehyde. To prepare tissues, dorsal root ganglia (DRG) were washed, dehydrated, cleared in xylene and then embedded in paraffin. Dorsal root ganglia were sectioned into 6 sections, mounted and stained with haematoxylin and eosin. Typically, between 80 to 100 sections were produced per DRG. Cellular dimensions were measured using a method adapted from Tomiwa et al. (1986) and Coggelshall et al (1990). A number was chosen at random between 1 and 9 to indicate the first section of the dorsal root ganglion to analyse. Perpendicular diameters of the cell body, nucleus and nucleolus of light-staining large-diameter nerve cells with clearly visible nucleoli were measured using an ocular micrometer and an oil immersion lens at 1000 times magnification. Measurements were then repeated in every tenth section at regular intervals throughout the dorsal root ganglion. Morphometric parameters from the 10 largest nerve cells in each dorsal root ganglion were averaged to provide cell body, nucleus and nucleolus diameter values for each animal.

### Sensory nerve conduction velocity (SNCV) determinations

SNCV was calculated from recordings of evoked H-plantar responses as previously described (McKeage et al, 1994). Briefly, animals were first lightly anaesthetised with intramuscular Hypnorm (Jansen Pharmaceuticals, Sydney, Australia) diluted 1:1 with sterilised water. Responses were evoked by electrically stimulating the sciatic nerve at the sciatic notch and the tibial nerve at the ankle of the left hind limb with percutaneous needle electrodes. H- and M-waves were recorded via a pair of superficial silver–silver chloride electrodes applied to the plantar and dorsal surfaces of the left hind limb. SNCV was calculated by dividing the distance between the stimulation sites at the sciatic notch and ankle by the difference in H-response latency after stimulation at the 2 sites.

### Statistical analysis

The statistical significance of differences between means was assessed using a *t*-test. The statistical significance of relationships between experimental parameters was assessed by linear and non-linear regression analysis using GraphPad Prism Version 3 (GraphPad Software, CA, USA). A two-sided $P$ value of $< 0.05$ was regarded as indicating statistical significance.

### RESULTS

### DRG morphology

Changes in DRG morphology have previously been documented in rats after treatment with multiple doses of cisplatin and other platinum drugs (Tomiwa et al, 1986; Muller et al, 1990; Cavaletti et al, 1992, 1998; Cece et al, 1995a, 1995b; Stacchiotti et al, 1995; Holmes et al, 1998). To determine the effects of a single dose of a platinum drug, DRG were collected from animals 24 hours after giving a injection of oxaliplatin of 10 mg $kg^{-1}$. The large diameter-light staining DRG nerve cells were the most affected and shrinkage of the nucleolus and cell body the most obvious changes (Figure 1).

### Time-course of DRG changes

To define the time-course of histologic toxicity after a single dose of oxaliplatin, DRG were collected for morphometry at 0, 2, 5, 8 and 24 hours, and at 2, 4, 7 and 14 days, after a single injection of 10 mg $kg^{-1}$. Control animals were given drug vehicle alone. Decreased nucleolar size was apparent within only a few hours of the treatment (Figure 2A) and nucleoli were maximally affected 24 to 48 hours after a single dose of oxaliplatin. Slow and partial recovery of nucleolar size began 48 hours after treatment. The nucleolus diameter was more affected than the nuclear and cell body dimensions by oxaliplatin (Figure 2B, C). Decreased nucleolar size was therefore the major quantitative change in DRG to be documented following a single dose of oxaliplatin and came on within a few hours of the treatment.

### Dose-dependence of DRG changes

To determine the dose-dependence of the DRG toxicity, animals were either untreated or treated with single doses of oxaliplatin (0.3, 1, 3, 10 and 30 mg $kg^{-1}$). DRG were collected 24 and 96 hours after treatment. Nucleolar size showed a non-linear dependence on dose at 24 hours ($r^2 = 0.99$) (Figure 3A) and again at 96 hours ($r^2 = 0.97$) (Figure 3B). Nuclear size showed a linear trend of dose-dependence at 96 hours ($r^2 = 0.70$, $P < 0.0001$, Figure 3C) but not at 24 hours. Cell body size also showed a linear trend of dose-dependence at 96 hours ($r^2 = 0.61$, $P < 0.0006$, Figure 3D) but not at 24 hours. Nucleolar and other DRG changes therefore depended on the dose of oxaliplatin.

© 2001 Cancer Research Campaign

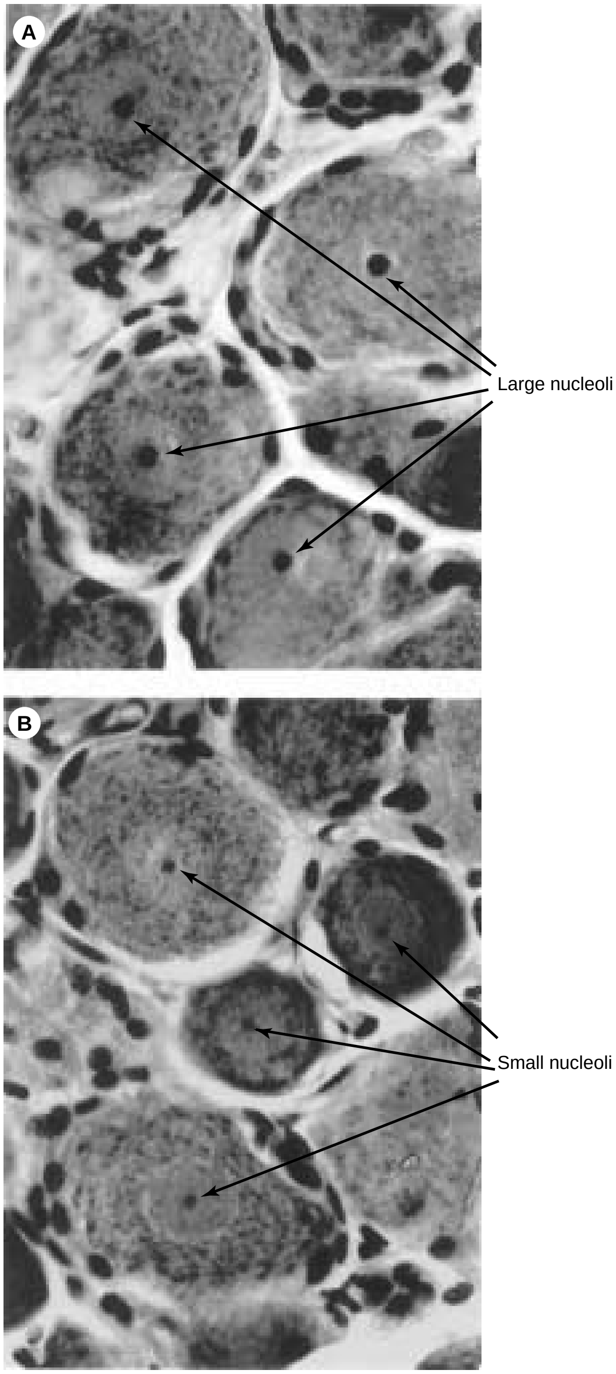

**Figure 1** Section of a dorsal root ganglion before (**A**) and 24 hours after oxaliplatin (**B**)

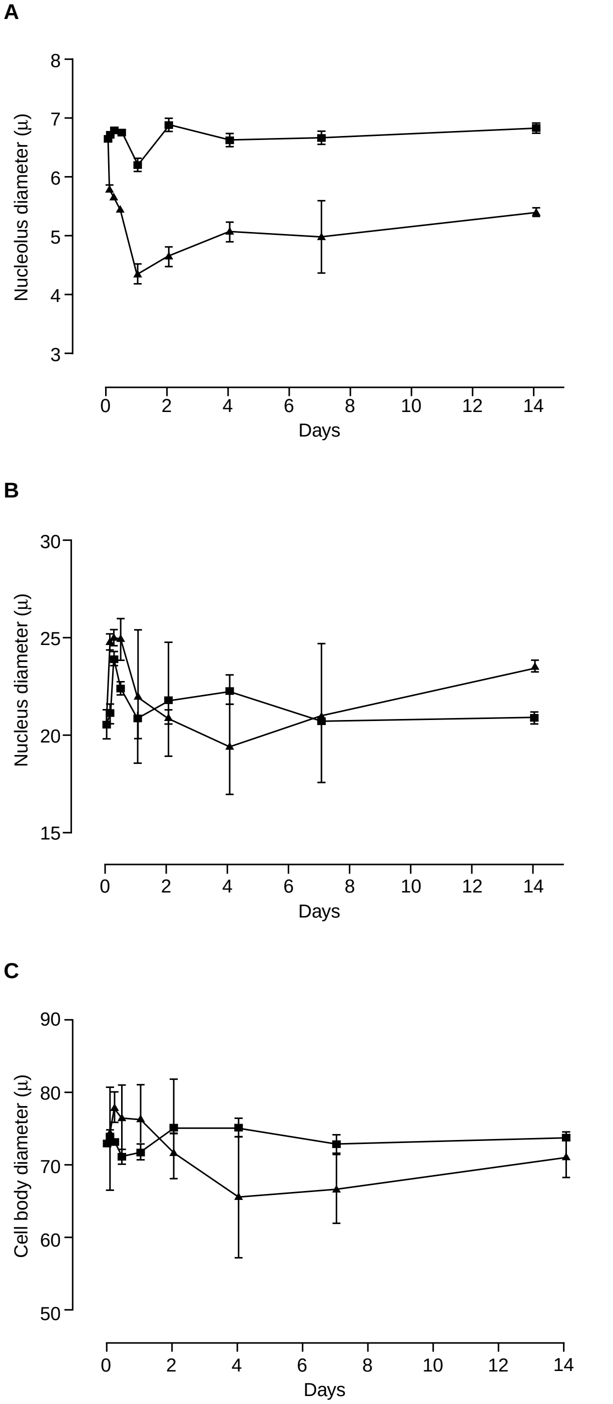

**Figure 2** Time-course of histological toxicity in DRG of rats after a single dose of oxaliplatin at 10 mg kg$^{-1}$ (25 µmol kg$^{-1}$). Nucleolar (**A**), nuclear (**B**) and cell body diameters (**C**) were determined at various times after oxaliplatin (▲) and drug vehicle alone (■). Symbols represent the mean and standard error of the mean of 3 to 4 animals

### Time-course of SNCV

To define the time-course of functional neurotoxicity after a single dose of oxaliplatin, SNCV was measured at 1, 4, 7, 14, 35, 56, 77, 98 and 119 days after a single injection of 10 mg kg$^{-1}$. The presence of toxicity was indicated by statistically significant differences in the mean SNCV values between the treatment and control groups. SNCV increased from 35 to 45 m s$^{-1}$ in the control group as the animals matured during the experiment. The onset of functional neurotoxicity was delayed until 14 days after the time of giving the single dose of oxaliplatin (Figure 4, Table 1). SNCV then slowly recovered so that by 120 days there was no difference in the mean SNCV values between the treatment and control groups. The onset of functional neurotoxicity was therefore delayed until well after the onset of DRG histological changes. Eventually there was complete recovery of SNCV.

© 2001 Cancer Research Campaign

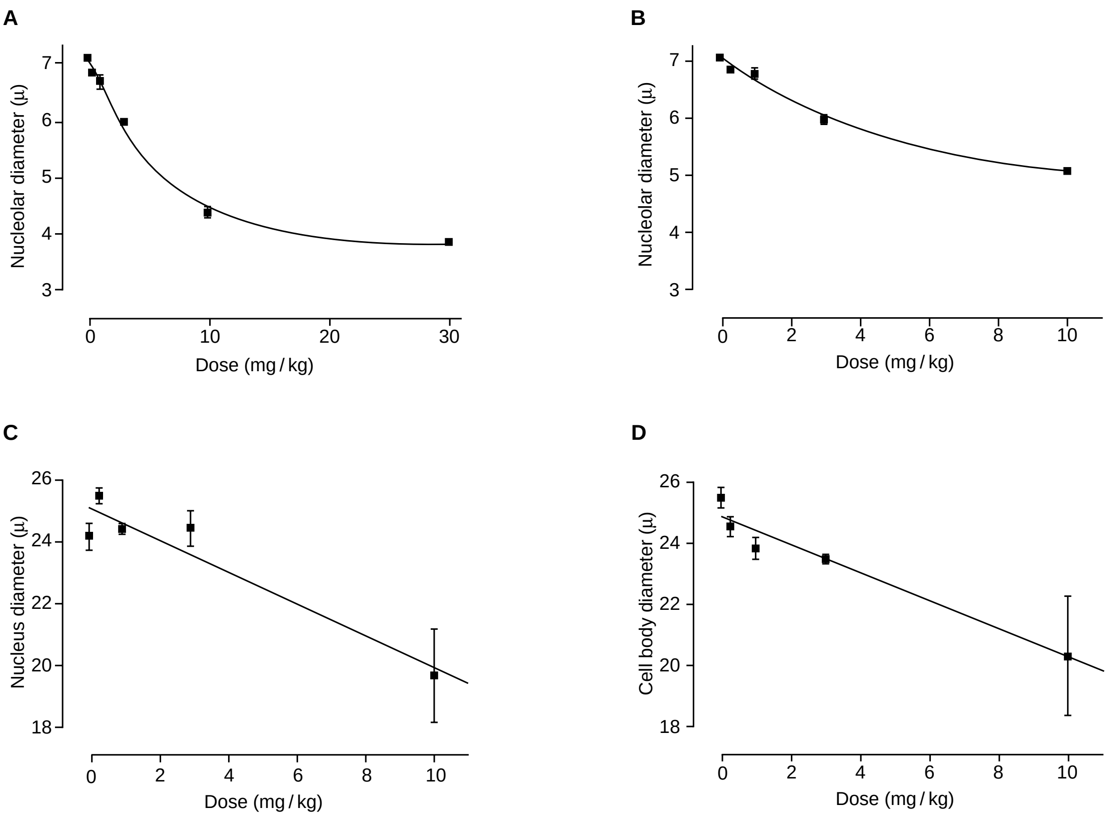

**Figure 3** Dose-dependent changes in DRG morphology. Nucleolar diameters were measured at 24 hours (**A**) and 96 hours (**B**) after single doses of oxaliplatin. Nuclear (**C**) and cell body (**D**) diameters were measured at 96 hours

**Table 1** Time-course of sensory nerve conduction velocity (SNCV) in rats after a single dose of oxaliplatin (10 mg $kg^{-1}$ or 25 µmol $kg^{-1}$). Control animals were given drug vehicle alone

| Time (days) | SNCV (Mean ± SD, *n* = 23–25) | | |
|---|---|---|---|
| | Control | Oxaliplatin | *P* |
| 0 | 37.0 ± 4.05 | 35.3 ± 3.17 | 0.1009 |
| 1 | 36.4 ± 5.39 | 34.9 ± 3.30 | 0.2340 |
| 4 | 37.6 ± 3.05 | 36.3 ± 3.78 | 0.2085 |
| 7 | 38.8 ± 4.82 | 36.8 ± 5.75 | 0.1453 |
| 14 | 41.3 ± 3.66 | 38.0 ± 3.88 | 0.0035 |
| 35 | 46.4 ± 3.31 | 43.1 ± 3.65 | 0.0021 |
| 56 | 46.4 ± 4.38 | 43.7 ± 3.70 | 0.0343 |
| 77 | 48.3 ± 3.72 | 46.4 ± 3.51 | 0.08 |
| 98 | 48.4 ± 3.94 | 44.8 ± 3.15 | 0.0045 |
| 119 | 48.4 ± 4.04 | 47.8 ± 4.31 | 0.42 |

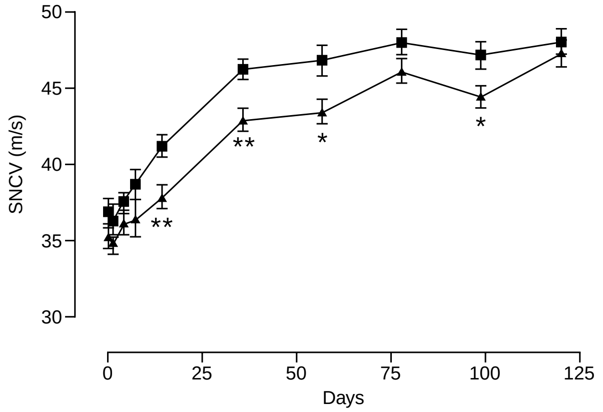

**Figure 4** Time-course of sensory nerve conduction velocity in rats given a single dose of oxaliplatin ((▲), 10 mg $kg^{-1}$ or 25 µmol $kg^{-1}$). Control animals were given drug vehicle alone (■). Symbols represent the mean and standard error of the mean (*n* = 23–25). **, $P < 0.005$; *, $P < 0.05$

### DRG changes during repeated-dose treatment

To determine the effect of multiple doses of different platinum drugs on DRG nucleolar size, animals were treated twice per week for 8 weeks with cisplatin, carboplatin, oxaliplatin, *R*,*R*-ormaplatin and *S*,*S*-ormaplatin at the maximum tolerated dose. DRG were collected after 14, 24, 36, 48 and 56 days of treatment. Time-dependent reductions in nucleolar size occurred with each of the different platinum treatments (Figure 5). The rate of change in nucleolar size varied (by 3-fold) among the different platinum drugs from –0.0169 to –0.0454 µ $day^{-1}$ (Table 2). Oxaliplatin caused a significantly faster rate of change in nucleolar size, and carboplatin caused a significantly slower rate of change, than cisplatin, carboplatin or *S*,*S*-ormaplatin (Table 3). Slower changes in the DRG nucleolus with carboplatin correspond with lower clinical neurotoxicity compared to other platinum drugs (Screnci et al, 2000). Repeated dose treatments of oxaliplatin and other platinum drugs were therefore associated with DRG nucleolar changes.

### Relationship between histological and functional toxicity

We have previously measured SNCV after 7, 14, 21, 28, 35, 42, 49 and 56 days of repeated dose treatment given twice per week for 8

© 2001 Cancer Research Campaign

**Table 2** Linear regression parameters of time-dependent changes in nucleolus size in dorsal root ganglia neurons

| | Cisplatin | Carboplatin | Oxaliplatin | *R,R*-ormaplatin | *S,S*-ormaplatin |
|---|---|---|---|---|---|
| Slope (µ $day^{-1}$) | – 0.02929 | – 0.016909 | – 0.04538 | – 0.03791 | – 0.03097 |
| Y-intercept (µ) | 4.73 | 4.82 | 4.69 | 4.61 | 4.69 |
| $r^2$ | 0.8785 | 0.5886 | 0.9441 | 0.8513 | 0.8929 |
| *P* | < 0.0001 | < 0.0001 | < 0.0001 | < 0.0001 | < 0.0001 |

**Table 3** Statistical significance (two-tailed *P* values) of differences in regression slopes for nucleolar change

| | Cisplatin | Carboplatin | Oxaliplatin | *R,R*-ormaplatin | *S,S*-ormaplatin |
|---|---|---|---|---|---|
| Cisplatin | – | 0.0007 | < 0.0001 | 0.0429 | 0.6131 |
| Carboplatin | | – | < 0.0001 | < 0.0001 | 0.0001 |
| Oxaliplatin | | | – | 0.0705 | < 0.0001 |
| *R,R*-ormaplatin | | | | – | 0.096 |
| *S,S*-ormaplatin | | | | | – |

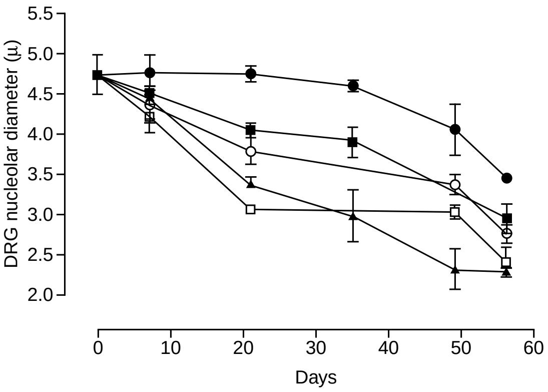

**Figure 5** Time-course of changes in nucleolar diameter of dorsal root ganglia of rats during administration of cisplatin 3.33 µmol $kg^{-1}$ (■), carboplatin 21.6 µmol $kg^{-1}$ (●), oxaliplatin 2.5 µmol $kg^{-1}$ (▲), *R, R*-ormaplatin 2.6 µmol $kg^{-1}$ (❑) and *S,S*-ormaplatin 2.6 µmol $kg^{-1}$ (○) repeated twice per week for 8 weeks. Error bars represent the standard error of the mean

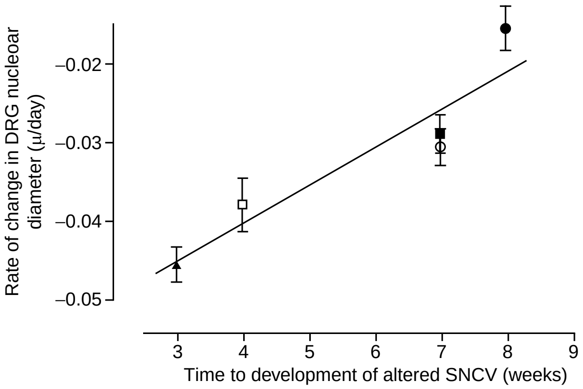

**Figure 6** Linear correlation between change in DRG nucleolar size and functional neurotoxicity ($r^2$ = 0.858; *P* = 0.0237). Symbols represent cisplatin (■), carboplatin (●), oxaliplatin (▲), *R,R*-ormaplatin (❑) and *S,S*-ormaplatin ○. Error bars are the standard error of the regression coefficient weeks with cisplatin, carboplatin, oxaliplatin, *R*,*R*-ormaplatin and *S*,*S*-ormaplatin at the same doses as used above (Screnci et al, 2000). To determine whether histological changes were related to functional neurotoxicity, SNCV determinations were correlated with DRG nucleolar size. A linear correlation was obtained between the rate of change in nucleolar size and the amount of treatment time taken for the development of altered SNCV ($r^2$ = 0.86; *P* = 0.02) (Figure 6). Platinum treatments causing faster changes in nucleoli size caused were more functional neurotoxicity than those causing slower nucleolar disturbance. Changes in nucleolar size were therefore linked with the functional neurotoxicity of platinum drugs.

### DISCUSSION

Oxaliplatin was a particular focus of our study because of the well-documented prominent neurotoxicity associated with the use of this platinum drug in the clinic (Extra et al, 1998; Misset, 1998). Acute neurotoxicity symptoms (distal paraesthesias and cold-related dysaesthesias) are common, appearing within a few hours of a single dose of oxaliplatin and lasting for up to 7 days. In the experiments described here, we demonstrate oxaliplatin-induced morphological changes in DRG with a similar time of onset. Following a single dose of oxaliplatin, changes in nucleolar size appeared within 2 hours. Nucleolar changes also showed a non-linear dependence on the dose of the platinum drug. Early nucleolar changes in DRG sensory nerve cells could possibly be the basis for the acute neurotoxicity symptoms that occur within a few hours of clinical infusions of oxaliplatin.

A peripheral sensory neuropathy may also develop clinically following repeated doses of oxaliplatin, and depend upon the cumulative dose (Extra et al, 1998; Misset, 1998). Neurotoxicity signs and symptoms are generally reversible and disappear over several months after the treatment has been completed. In the rat model, the changes in DRG morphology are only slowly reversible, suggesting that repeated dosing would give rise to cumulative toxicity. This is evident in the changes in SNCV, which were delayed in onset until at least 14 days after treatment. Functional neurotoxicity induced by oxaliplatin eventually recovered several months after a single dose of the drug while morphological changes persisted. The pattern of experimental toxicity therefore appears to reflect the various manifestations of the

© 2001 Cancer Research Campaign neurotoxicity associated with oxaliplatin in the clinical setting, particularly its acute onset, development of cumulative sensory neuropathy and functional reversibility.

Previous studies of DRG morphology have consistently described the nucleolus of sensory nerve cells as being among the most frequently and severely affected structures in the peripheral nervous system by platinum drugs (Tomiwa et al, 1986; Muller et al, 1990; Cavaletti et al, 1992, 1998; Cece et al, 1995a, 1995b; Stacchiotti et al, 1995; Holmes et al, 1998). The present findings strengthen the association between nucleolar changes in DRG sensory nerve cells and the neurotoxicity for a series of platinum drugs with differing degrees of associated neurotoxicity. When different platinum drugs were given repeatedly over several weeks, their neurotoxicity correlated with the rapidity of change in nucleolar size. Thus, the findings presented above provide further evidence that the nucleolus of the DRG sensory nerve cell is both an initial and critical target within the peripheral nervous system for platinum drugs.

Nucleolar changes have also been associated with other forms of platinum toxicity. Nucleoli of renal tubular epithelial cells of rats become highly condensed and segregated into fibrillary and granular components prior to the onset of many of the other structural and functional changes that accompany cisplatin-induced renal toxicity (Lehane et al, 1979; Jones et al, 1985). In primary cultures of rat renal proximal tubule cells, exposure to cisplatin results in nucleolar disintegration, disruption of the rough endoplasmic reticulum and inhibition of protein synthesis (Leibbrandt et al, 1995). Interference with nucleolar function may therefore be the basis for several of the side effects associated with the clinical use of platinum drugs.

Nucleolar shrinkage and disintegration are recognised as typical changes occurring as a result of transcriptional arrest (Brasch, 1990). The nucleolus is normally a very prominent structure in DRG sensory nerve cells (Schwatz, 1991), presumably because of the substantial amounts of rRNA and protein synthesis occurring within these cells (Shaw and Jordan, 1995).

Changes in nucleolar size induced by platinum drugs in DRG sensory nerve cells could therefore be accounted for by the inhibition of rRNA synthesis, which is a known effect of platinum-based antitumour drugs (Jordon and Carmo-Fonseca, 1998).

The mechanism of such inhibition is not yet clearly established. DNA-adducts formed by cisplatin can cause premature termination of transcription at sites of 1,2-intrastrand d(GG) and d(AG) cross-links, thus directly blocking the progress of RNA polymerase I along the DNA template (Lemaire et al, 1991). An alternative mechanism (Treiber et al, 1994; Vichi et al, 1997; Zhai et al, 1998) is that 1,2-intrastrand d(GG) adducts sequester nucleolar transcription factors human upstream binding factor (hUBF) and TATA box-binding protein (TBP), thereby reducing the amount of transcription factors available for initiation of rDNA transcription HeLa cells exposed to cisplatin exhibit a redistribution of nucleolar transcription factors (Jordan and Carmo-Fonseca, 1998). However, although such effects can be demonstrated in vitro, the number of platinum DNA adducts associated with clinical treatment is of the order of 400 per genome (Poirer et al, 1992). The frequency of adducts (about one adduct per 15 million base pairs) may not be sufficient to block rDNA transcription directly. Furthermore, since there are a number of copies of tandemly repeated rDNA and presumably an even higher number of transcription factors, it is questionable whether the decoy mechanism could account for the magnitude of the effects observed. Furthermore, if both cytotoxicity and neurotoxicity are the result of the same types of DNA adduct, a mechanism must be provided for the apparent dissociation of antitumour and neurotoxic effects.

Our previous study (Screnci et al, 2000), using a similar series of drugs, demonstrated that neurotoxicity did not correlate with peripheral nerve accumulation, nor with drug lipophilicity. However, serum protein reactivity correlated with neurotoxicity, in both rats (r = 0.89; $P = 0.0005$) and patients (r = 0.99; $P = 0.0002$). Consideration might therefore be given to the possibility that reaction with intracellular proteins is important for inhibition of rRNA synthesis. Cisplatin is known to react with unusual sites in proteins (Ivanov et al, 1998) and could, for example, affect rRNA synthesis through binding directly to transcription factors. Whatever the exact mechanism, disruption of rRNA synthesis could be the basis for the changes in nucleolar structure in DRG sensory nerve cells of rats treated with platinum drug described here and elsewhere.

In summary, reduction in nucleolar size is the major quantitative histological change in DRG sensory nerve cells of rats after treatment with platinum drugs. Changes in the size of the nucleoli of DRG sensory nerve cells are also dose-related, occur early after treatment and correlate with the development of functional neurotoxicity as measured by altered SNCV. Measurement of DRG nucleolar size may therefore have utility in assessing the neurotoxic potential of new platinum drugs. The method could also be applied ex vivo using short-term cultures of DRG but validation of the in vitro system will first be required. Nucleolar dimensions could also be a useful endpoint for studies of strategies to lessen neurotoxicity. The results of this study are consistent with a model of neurotoxicity where the arrest of rRNA synthesis leads to reduced protein synthesis and reduced function of DRG sensory nerve cells.

### ACKNOWLEDGEMENT

This project was funded by the Cancer Society of New Zealand. The Auckland Medical Research Foundation provided a scholarship for D Screnci.

### REFERENCES

Brasch K (1990) Drug and metabolite-induced perturbations in nuclear structure and function: a review. *Biochem Cell Biol* **68**: 408–426

Cavaletti G, Tredici G, Marmiroli P, Petruccioloi MG, Barajon I and Fabbrica D (1992) Morphometric study of the sensory neuron and peripheral nerve changes induced by chronic cisplatin (DDP) administration in rats. *Acta Neuropathol* **84**: 364–371

Cavaletti G, Fabbrica D, Minoia C, Frattola L and Tredici G (1998) Carboplatin toxic effects on the peripheral nervous system of the rat. *Ann Oncol* **9**: 443–447

Cece R, Petruccioli MG, Cavaletti G, Barajon I and Tredici G (1995a) An ultrastructural study of neuronal changes in dorsal root ganglia (DRG) of rats after chronic cisplatin administrations. *Histol Histopathol* **10**: 837–845

Cece R, Petruccioli MG, Pizzini G, Cavaletti G and Tredici G (1995b) Ultrastructural aspects of DRG satellite cell involvement in experimental cisplatin neuronopathy. *Submicrosc Cytol Pathol* **27**: 417–425

Coggeshall RE, La Forte R and Klen CM (1990) Calibration of methods for determining numbers of dorsal root ganglia cells. *J Neurosci Meth* **35**: 187–194

Einhorn LH (1997) Testicular Cancer: An Oncological success Story. *Clin Cancer Res* **3**: 2630–2632

Extra J-M, Marty M, Brienza S and Misset J-L (1998) Pharmacokinetics and safety profile of oxaliplatin. *Semin Oncol* **25(**Suppl 5**)**: 13–22

Fink D and Howell SB (2000) How does cisplatin kill cells? In: *Platinum-based drugs in cancer therapy*, Kelland LR and Farrell N (eds.) pp. 149–167. Humana Press: Totowa NJ

Gregg RW, Molepa JM, Monpetit VJA, Mikeal NZ, Redmond D, Gadia M and Stewart DJ (1992) Cisplatin neurotoxicity: the relationships between dosage,

 *© 2001 Cancer Research Campaign*

time and platinum concentration in neurological tissues, and morphological evidence of toxicity. *J Clin Oncol* **10**: 795–803

Harder HC and Rosenberg B (1970) Inhibitory effects of antitumor platinum compounds on DNA, RNA and protein synthesis in mammalian cells *in vitro. Int J Cancer* **6**: 207–216

Holmes J, Stanko J, Varchenko M, Ding H, Madden VJ, Bagnell CR, Wyrick SD and Chaney SG (1998) Comparative neurotoxicity of oxaliplatin, cisplatin and ormaplatin in a Wistar rat model. *Toxicol Sci* **46**: 342–351

Ivanov AI, Christodoulou J, Parkinson JA, Barnham KJ, Tucker A, Woodrow J and Sadler PJ (1998) Cisplatin binding sites on human albumin. *J Biol Chem* **273**: 14721–14730

Jamieson ER and Lippard SJ (1999) Structure, recognition and processing of cisplatin-DNA adducts. *Chem Rev* **99**: 2467–2498

Jones TW, Chopra S, Kaufman JS, Flamenbaum W and Trump BF (1985) *cis*-diamminedichloroplatinum(II)-induced acute renal failure in the rat. *Lab Invest* **52**: 363–374

Jordon P and Carmo-Fonseca M (1998) Cisplatin inhibits synthesis of ribosomal RNA *in vivo. Nucleic Acids Res* **26**: 2831–2836

Lehane D, Winston A, Gray R and Daskal Y (1979) The effect of diuretic pre-treatment on clinical, morphological and ultrastructural *cis*-platinum induced nephrotoxicity. *Int J Radiat Oncol* **5**: 1393–1399

Leibbrandt MEI, Wolfgang GHI, Metz AL, Ozobia AA and Haskins JR (1995) Critical subcellular targets of cisplatin and related platinum analogs in rat renal proximal tubule cells. *Kidney Int* **48**: 761–770

Lemaire M-A, Schwartz A, Rahmount AR and Leng M (1991) Interstrand cross-links are preferentially formed at the d(GC) sites in the reaction between cis-diamminedichloroplatinum(II) and DNA. *Proc Natl Acad Sci USA* **88**: 1982–1985

McKeage MJ (2000) Clinical toxicology of platinum-based cancer chemotherapeutic agents. In: *Platinum-based drugs in cancer therapy*, Kelland LR and Farrell N (eds.) pp. 251–275 Humana Press: Totowa NJ

McKeage MJ, Boxall F, Jones M and Harrap KR (1994) Lack of neurotoxicity of oral bis-acetato-ammine-dichloro-cyclohexylamine-platinum(IV) (JM216) in comparison to cisplatin and tetraplatin in the rat. *Cancer Res* **54**: 629–631

Misset J-L (1998) Oxaliplatin in practice. *Brit J Cancer* **77 (**Suppl 4**)**: 4–7

Muller LJ, Gerritsen Van Der Hoop R, Moorer-Van Delft CM, Gispen WH and Roubos EW (1990) Morphological and electrophysiological study of the effects of cisplatin and ORG2766 on rat spinal ganglia neurons. *Cancer Res* **50**: 2437–2442

O'Rourke TJ, Weiss GR, New P, Burris HA, Rodriguez G, Eckhart J, Hardy J, Kuhn JG, Fields S and Clark GM (1994) Phase I clinical trial of ormaplatin (tetraplatin, NSC363812) *Anti-Cancer Drug* **5**: 520–526

Ozols RF (2000) Optimum chemotherapy for ovarian cancer. *Int J Gynecol Cancer* **10(**Suppl 1**)**: 33–37

Ozols RF, Corden BJ, Jacob J, Wesley MN, Ostchega Y and Young RC (1984) High-dose cisplatin in hypertonic saline. *Ann Intern Med* **100**: 19–24

Poirier MC, Reed E, Litterest CL and Gupta-Burt S (1992) Persistence of platinum-amine-DNA adducts in gonads and kidneys of rats and multiple tissues from cancer patients. *Cancer Res* **52**: 149–153

Schilder RJ, La Creta FP, Perez RP, Johnson SW, Brennan JM, Rogatko A, Nash S, McAleer C, Hamilton TC, Rody D, Young RC, Ozol RF and O'Dwyer PJ (1994) Phase I and pharmacokinetic study of ormaplatin (tetraplatin, NSC363812) administered on a day 1 and 8 schedule. *Cancer Res* **54**: 709–717

Schwartz JH (1991) The Cytology of Neurons In: *Principles of Neural Science (3rd Ed.)* Kandel ER, Schwartz JH and Jessell TM (eds.), pps 37–48. Prentice-Hall International Ltd: London

Screnci D, Er HM, Hambley TW, Galettis P, Brouwer W and McKeage MJ (1997) Stereoselective peripheral sensory neurotoxicity of diaminocyclohexane platinum enantiomers related to ormaplatin and oxaliplatin. *Brit J Cancer* **76**: 502–510

Screnci D, McKeage MJ, Galettis P, Hambley TW, Palmer BD and Baguley BC (2000) Relationships between hydrophobicity, reactivity, accumulation and peripheral nerve toxicity of a series of platinum drugs. *Brit J Cancer* **82**: 966–972

Shaw PJ and Jordon EG (1995) The Nucleolus. *Annu Rev Cell Dev Bi* **11**: 93–121

Sorenson CM, Barry MA and Eastman A (1992) Analysis of events associated with cell cycle arrest at G2 phase and cell death induced by cisplatin. *J Natl Cancer* 182: 749–755

Stacchiotti A, Rezzani R, Rodella L and Ventura RG (1995) Lysosomal changes in rat spinal ganglia neurons after prolonged treatment with cisplatin. *Acta Anat* **153**: 236–242

Thomas GM (1999) Improved treatment for cervical cancer-concurrent chemotherapy and radiotherapy. *New Engl J Med* **340**: 1198–1199

Thompson SW, Davis LE, Kornfeld M, Hilger DR and Standefer JC (1984) Cisplatin neurotoxicity; clinical, electrophysiologic, morphologic and toxicologic studies. *Cancer* **54**: 1269–1275

Tomiwa K, Nolan C and Cavanagh JB (1986) The effects of cisplatin on rat spinal ganglia: a study by light and electron microscopy and morphometry. *Acta Neuropathol* **69**: 295–308

Treiber DK, Zhai X, Jantzen H-M and Essigmann JM (1994) Cisplatin-DNA adducts are molecular decoys for the ribosomal RNA transcription factor hUBF (human upstream binding factor). *P Natl Acad Sci USA* **91**: 5672–5676

Vichi P, Coin F, Renaud J-P, Vermeulen W, Hoeijmakers JHJ, Moras D and Egly J-M (1997) Cisplatin- and UV-damaged DNA lure the basal transcription factor TFIID/TBP. *EMBO J* **16**: 7444–7456

Zhai X, Beckmann H, Jantzen H-M and Essigmann JM (1998) Cisplatin-DNA adducts inhibit Ribosomal RNA synthesis by high-jacking the transcription factor human upstream binding factor. *Biochemistry-US* **37**: 16307–16315

*© 2001 Cancer Research Campaign*
